# DeepRank-GNN: a graph neural network framework to learn patterns in protein–protein interfaces

**DOI:** 10.1093/bioinformatics/btac759

**Published:** 2022-11-24

**Authors:** Manon Réau, Nicolas Renaud, Li C Xue, Alexandre M J J Bonvin

**Affiliations:** Computational Structural Biology Group, Department of Chemistry, Bijvoet Centre, Faculty of Science, Utrecht University, Utrecht 3584CH, The Netherlands; Netherlands eScience Center, Amsterdam 1098 XG, The Netherlands; Center for Molecular and Biomolecular Informatics, Radboudumc, Nijmegen 6525 GA, The Netherlands; Computational Structural Biology Group, Department of Chemistry, Bijvoet Centre, Faculty of Science, Utrecht University, Utrecht 3584CH, The Netherlands

## Abstract

**Motivation:**

Gaining structural insights into the protein–protein interactome is essential to understand biological phenomena and extract knowledge for rational drug design or protein engineering. We have previously developed DeepRank, a deep-learning framework to facilitate pattern learning from protein–protein interfaces using convolutional neural network (CNN) approaches. However, CNN is not rotation invariant and data augmentation is required to desensitize the network to the input data orientation which dramatically impairs the computation performance. Representing protein–protein complexes as atomic- or residue-scale rotation invariant graphs instead enables using graph neural networks (GNN) approaches, bypassing those limitations.

**Results:**

We have developed DeepRank-GNN, a framework that converts protein–protein interfaces from PDB 3D coordinates files into graphs that are further provided to a pre-defined or user-defined GNN architecture to learn problem-specific interaction patterns. DeepRank-GNN is designed to be highly modularizable, easily customized and is wrapped into a user-friendly python3 package. Here, we showcase DeepRank-GNN’s performance on two applications using a dedicated graph interaction neural network: (i) the scoring of docking poses and (ii) the discriminating of biological and crystal interfaces. In addition to the highly competitive performance obtained in those tasks as compared to state-of-the-art methods, we show a significant improvement in speed and storage requirement using DeepRank-GNN as compared to DeepRank.

**Availability and implementation:**

DeepRank-GNN is freely available from https://github.com/DeepRank/DeepRank-GNN.

**Supplementary information:**

Supplementary data are available at *Bioinformatics* online.

## 1 Introduction

Protein–protein interactions (PPIs) are essential in all cellular processes of living organisms including cell growth, structure, communication, protection and death. Adding the structural dimension to PPI is fundamental to understand normal and altered physiological processes and to propose solutions to restore them. In the past decades, a large number of isolated protein and PPI structures have been solved by experimental approaches (e.g. X-ray crystallography, nuclear magnetic resonance and cryogenic electron microscopy). The diversity and quantity of structural data recently enabled treating PPI data with machine learning approaches that were previously devoted to small molecule toxicity (Mayr *et al.*, 2016), affinity (Jiménez *et al.*, 2018; Karlov *et al.*, 2020; Ragoza *et al.*, 2017; Son and Kim, 2021) and binding mode (Francoeur *et al.*, 2020; Morrone *et al.*, 2020; Torng and Altman, 2019) prediction.

Given the remarkable success of convolutional neural network (CNN) in retrieving patterns in images (Krizhevsky *et al.*, 2017), CNN approaches have been developed to learn interaction patterns in PPI interfaces (Renaud *et al.*, 2021; Wang *et al.*, 2020) or to assess the quality of protein structures (Baldassarre *et al.*, 2021; Pagès *et al.*, 2019). The uniqueness of each approach originates from the designed network architecture and importantly, the data representation and resolution. An example is MASIF (Gainza *et al.*, 2020) that makes use of a high-level representation of proteins, focusing on their surface described as an ensemble of overlapping patches. The patches are fed into different CNNs in order to build relevant fingerprints that can be further used for ultra-fast interaction prediction tasks based on the complementarity or the similarity of the fingerprints. DOVE (Wang *et al.*, 2020) evaluates protein–protein docking models using a 3D-CNN approach on a higher resolution—atomic-level—representation of the interface mapped into a 3D grid. Although no exhaustive benchmark exists with state-of-the-art approaches, both tools display high performance on the benchmark set used for their evaluation and hold the promise to improve over time with the availability of new data and the improvement of data storage and computation power. As the recent major advances made by Alphafold2 in predicting protein structures (Jumper *et al.*, 2021) and protein multimeric states are likely to lead to an exponential generation of multimers over years, including true and false partners, the availability of reliable quality assessment tools should become a strong ally to reach the ambitious objective of modeling of the entire interactome.

We have recently developed DeepRank (https://github.com/DeepRank/deeprank), an open-source configurable deep-learning framework wrapped into a user-friendly python3 package (Renaud *et al.*, 2020, 2021). DeepRank maps atomic and residue-level features from PPI interfaces to 3D grids and applies a customizable 3D CNN pipeline to learn problem-specific interaction patterns. DeepRank was applied to two problems where it competed with- or outperformed state-of-the-art methods, including a machine learning-based model, iScore (Geng *et al.*, 2020) that makes use of a graph representation and the classical energy-based scoring function implemented in HADDOCK (van Zundert *et al.*, 2016).

CNNs however come with limitations: first, they are sensitive to the input PPI interface orientation which may require data augmentation (i.e. multiple rotations of the input data) for the network to provide consistent predictions regardless of the orientation of the PPI; second, the size of the 3D grid is pre-defined for all input data in DeepRank 0.2.0, which does not reflect the variety in interface sizes observed in experimental structures and may be problematic for large interfaces that do not fit inside the pre-defined grid size.

A solution to this problem is to use a graph representation of PPI interface. A graph is defined as an ensemble of nodes (e.g. atoms and residues) and edges (e.g. covalent bond and contacts), and is often represented with a feature matrix containing attributes assigned to each node of the graph, and an adjacency matrix—or edge matrix—describing the connectivity between the nodes. A graph neural network (GNN) iteratively updates a node’s features integrating the node’s neighborhood information (an operation called message passing). GNNs can be trained to learn the optimal updated node features to predict the properties of a single protein or a complex of proteins (Cao and Shen, 2020; Igashov *et al.*, 2021; Wang *et al.*, 2021). Contrary to CNNs, the convolution operations on graphs can be independent from Cartesian coordinates and only rely on the relative local connectivity between nodes, therefore making graphs rotational invariant. GNNs are also invariant with respect to the ordering of nodes in the feature and adjacency matrices, and the network can accept any size of graph, therefore more naturally representing the diversity of PPIs. Based on these arguments, different GNN-based tools have been designed for PPI site prediction (Fout *et al.*, 2017; Mahbub and Bayzid, 2022) and to assess the quality of protein–protein complexes (Wang *et al.*, 2021). An example of the latter is the GNN version of DOVE (DOVE-GNN) that demonstrated significant improvement in the docking models classification task over the CNN version (Wang *et al.*, 2021).

Building up on our previous framework DeepRank (CNN based), we present here DeepRank-GNN (Réau and Renaud, 2021), a versatile software that takes advantage of the intrinsic properties of graph representation and graph convolutions. DeepRank-GNN converts PPI interfaces from 3D coordinates PDB files into graphs and enables the application of pre-defined or user-defined GNN architectures to train a network to make predictions related to the properties of PPI interfaces, such as the quality of a docking model or the likelihood that a given interface is biologically relevant. DeepRank-GNN can automatically compute docking-specific target values when reference PDB files are provided or assign user-provided target values to the graphs. We describe the main functionalities of DeepRank-GNN and showcase its application to the scoring of docking models and the discrimination of biological and crystal interfaces. Detailed documentation is available online at https://deeprank-gnn.readthedocs.io/.

## 2 Materials and methods

### 2.1 DeepRank-GNN overview

**Fig. 1.**
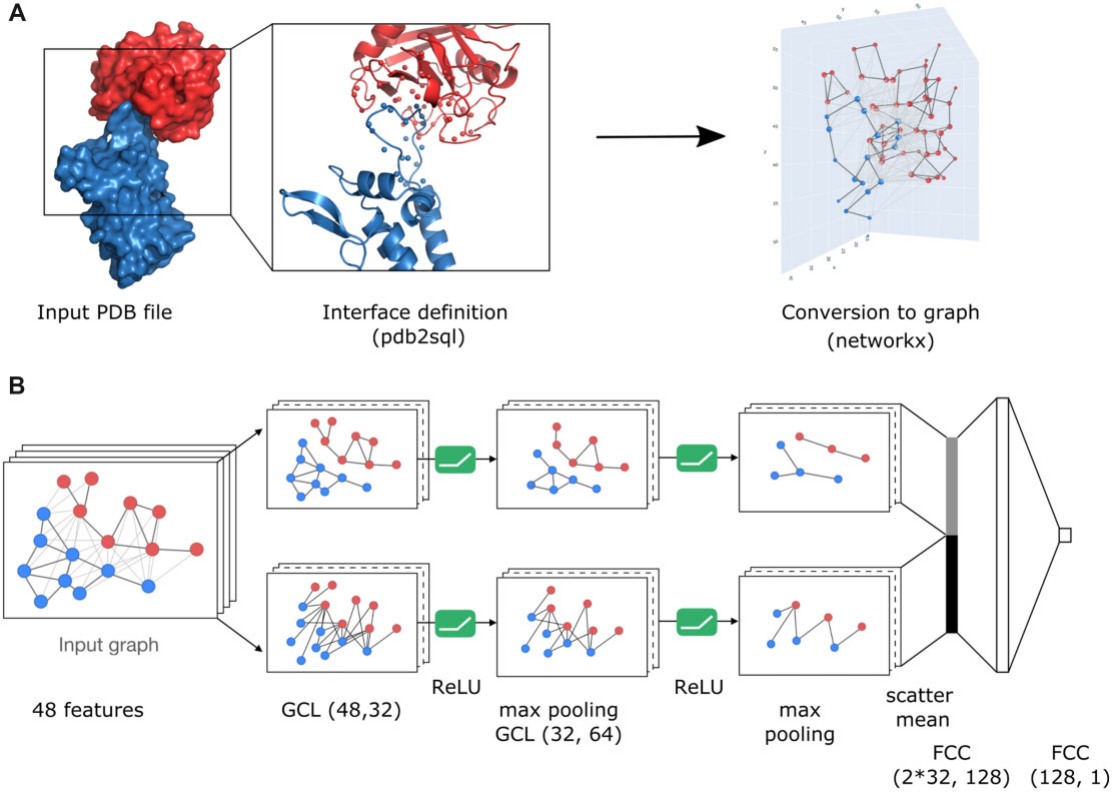
Overview of the DeepRank-GNN framework. (**A**) DeepRank-GNN identifies interface residues and converts them into an interface graph. Internal edges are defined between residues from the same chain having heavy atoms within a 3 Å distance cutoff from each other, while external edges are defined between residues from different chains having heavy atoms within the 8.5 Å cutoff. (**B**) *Example of GNN architecture* (GINet). The graph representation of a PPI is split into two sub-graphs, i.e. the internal graph connecting atoms from the same protein and the external graph connecting atoms from distinct proteins. The two sub-graphs are sequentially passed to two consecutive convolution/activation/pooling layers. The two final graph representations are flattened using the mean value of each feature and merged before applying two fully connected layers. GCL, graph convolution layer; FCC, fully connected layer

DeepRank-GNN is a python3 package that offers a complete framework to learn PPI interface patterns in an end-to-end fashion using GNN. The overall design of DeepRank-GNN was inherited from our previous package DeepRank that focuses on the scoring PPI using 3dcnn neural networks and consists of two mains parts (Fig. 1) (i) the conversion of 3D PPI interfaces into interaction graphs with node and edge features using the networkx (Hagberg *et al.*, 2008) and pdb2sql (Renaud and Geng, 2021b) packages and (ii) the training and the evaluation of a graph neural network model using PyTorch geometric (Paszke *et al.*, 2017). An overview of the software architecture is provided in Supplementary Figure S1. We briefly present both parts below and refer the reader to the online documentation for further information (https://deeprank-gnn.readthedocs.io/).

#### 2.1.1 Graph generation

DeepRank-GNN converts PPI interfaces into residue-level graphs (Fig. 1A). It takes PDB 3D coordinate files as an input and defines the interface between two chains using pdb2sql (Renaud and Geng, 2021b), our PDB file parser using a structured query language (SQL). By default, the interface is defined by all the residues involved in intermolecular contacts, i.e. the residues of a given chain having a heavy atom within an 8.5 Å distance cutoff of any heavy atom from another chain. These contact residues form the nodes of the graph. *Interface* edges are defined between two contact residues from distinct chains presenting a minimal atomic distance smaller than 8.5 Å. In addition, *internal* edges are defined between two contact residues of the same chain provided they have heavy atoms within 3 Å from each other. These default distance cut-offs can be tailored by the user. The types of edges can be later considered to perform different convolution operations on the graph.

The graphs are stored in HDF5 format that is suited for large dataset storage and allows efficient memory usage and fast input/output operations during the network training.

#### 2.1.2 Featurization

By default, DeepRank-GNN computes and assigns an ensemble of residue-level features to each node. Those are summarized in Table 1. The feature computation can rapidly become a limiting step if the computation speed is not optimized. In DeepRank-GNN, the assignment of residue type, charge, polarity and buried surface area features are considerably faster than the computation of the residue depth, i.e. the average distance of the atoms of a residue from the solvent accessible surface, and the half sphere exposure. Provided that the information brought by the two latter could implicitly be deduced from the buried surface area feature and the node environment, they are not calculated by default for the sake of time efficiency. Pre-computed position-specific scoring matrices (PSSM) are required for the assignment of PSSM-related features. We advise querying a dataset of pre-computed PSSM matrices such as the 3DCONS (http://3dcons.cnb.csic.es/), the Conserved Domains Database (Lu *et al.*, 2020) or using our in-house PSSM generation tool PSSMGen (Renaud and Geng, 2021a).

**Table 1.** Features computed in DeepRank-GNN

Name of features	Full name	Description	Default	Number of parameters	Type
Type	Residue type	One-hot encoded	Default	20	Node feature
Charge	Residue charge		Default	1	Node feature
Polarity	Residue polarity	One-hot encoded	Default	4	Node feature
BSA	Buried surface area	FreeSASA	Default	1	Node feature
PSSM	Position-specific scoring matrix		Optional	20	Node feature
Cons	Conservation score—from PSSM		Optional	1	Node feature
ic	Information content—from PSSM		Optional	1	Node feature
Depth	Residue depth	MSMS—Biopython	Optional	1	Node feature
hse	Residue half sphere exposure	Biopython	Optional	1	Node feature
Distance	Normalized distance		Default	1	Edge feature

To encode the relative positions of the nodes in the graph, and therefore the overall structure of the interface, we assign a distance feature to the internal and external edges. This distance feature is based on the smallest atomic distance between two residues (nodes) that is transformed into an interaction strength by equation 1.

(1) $$e_{ij}=\;\tan\;h\left(-\frac{x}{2}+2\right)+1\#,$$

where *x* is the smallest distance between two residues (Å).

The interaction strength ranges from 0 for long distances to 1.96 for null distances and provides a normalized feature of the internode distance. While other normalization functions could be used, we believe that the careful exploration regarding the influence of the normalization function on the performance of the training process is out of the scope of this manuscript.

#### 2.1.3 Target assignment

Many different metrics have been developed to quantify the relevance of PPI interfaces and can be used as target values during the network training and evaluation phases. In a docking scenario where the goal is to identify near-native models, the user can provide a reference structure, i.e. the experimentally solved bound conformation of the complex, for DeepRank-GNN to automatically compute target values in the pre-processing stage based on CAPRI quality criteria (Lensink *et al.*, 2007) (Supplementary Table S1). For other applications and/or use cases, a reference structure is not required and users may input their own problem-specific target values or develop new metric calculations and integrate these metrics in the computational workflow.

#### 2.1.4 Model training/evaluation/test

All the prerequisites to train and to evaluate a GNN model are detailed in our online documentation. The user can run DeepRank-GNN in a regression or classification mode. The loss function is automatically set to mean square error (MSE) for regression tasks or to cross-entropy for classification tasks. Weights can be assigned to classes to balance the cross-entropy loss calculation in case of an imbalanced dataset. We also propose an automated weight computation that assigns weights inversely proportional to each class representation in the training set for classification tasks on imbalanced datasets (see Supplementary Information).

#### 2.1.5 Network

DeepRank-GNN provides a flexible structure allowing users to define their own network architectures or use pre-defined ones (see the online documentation).

#### 2.1.6 Quality metrics

DeepRank-GNN provides tools to swiftly compute the quality metrics summarized in our online documentation (https://deeprank-gnn.readthedocs.io/en/latest/tutorial.train_model.html#analysis). Upon the definition of a threshold value to binarize the data, all classification metrics can be applied to continuous targets and prediction values.

### 2.2 Application 1—the scoring of docking models

We evaluated DeepRank-GNN’s performance as a docking model scoring tool. We designed a GNN architecture that was trained and evaluated on the Docking Benchmark version 5 (BM5) dataset and further tested on an external set, the CAPRI scoreset.

#### 2.2.1 BM5 benchmark

The BM5 dataset has been designed for docking purposes and encompasses a non-redundant set of 231 complexes for which the individual structure of interacting proteins is available in a bound and an unbound conformation (Vreven *et al.*, 2015). We discarded the 56 antibody–antigen complexes plus the complexes involving more than two chains and worked on the remaining 142 dimers. As described in Renaud *et al.* (2021), we generated 25 300 models per complex using our integrative modeling software HADDOCK (see Supplementary Information). The overall dataset comprises 3 592 600 models and is available from the SBGrid data repository https://data.sbgrid.org/dataset/843/.

We performed 10-fold cross-validation in which the training and the evaluation sets change over the folds while the test set remains constant. The test set consists of all docking models generated for 15 randomly selected complexes (379 500 models, 10% of the dataset, see Supplementary Table S2). Each fold consists of 10% of the docking models per remaining complex without any models overlap between folds. Their composition preserves the distribution of CAPRI iRMSD classes (Lensink *et al.*, 2007) (reporting on the quality of the models) per complex. This was achieved using sklearn StratifiedKFold tool. The 127 complexes not included in the test set are then split into a training (80%, i.e. 102 complexes, 258 060 models) and an evaluation set (20%, i.e. 25 complexes, 63 250 models per fold). The detailed content of each fold’s training and evaluation set is provided in our GitHub repository. A number of complexes displaying important clashes could not be converted into graphs.

#### 2.2.2 CAPRI benchmark

The CAPRI score set (Lensink and Wodak, 2014) was used as an external test set. It consists of 13 protein dimers for a total of 16 666 models generated by over 40 different research teams using a variety of software. It is acknowledged as the most diverse set of docking models with targets of different complexity.

The HADDOCK (van Zundert *et al.*, 2016), iScore (Geng *et al.*, 2020), DOVE (Wang *et al.*, 2020) and DeepRank scores were computed on the CAPRI score set as described in Renaud *et al.*, 2021. Deeprank and DOVE are two CNN-based scoring approaches, iScore is graph-kernel based, and HADDOCK uses a classic scoring function that consists of a linear combination of energy terms (see Supplementary Information). These scores for the BM5 and CAPRI score sets were obtained from the DeepRank paper (Renaud *et al.*, 2021) and can be downloaded from: https://data.sbgrid.org/dataset/843/.

#### 2.2.3 Graph generation and target value computation

PPI interfaces were converted into graphs using the default DeepRank-GNN parameters, default nodes features (residue type, polarity, charge and BSA) and edge feature (distance) and additional PSSM information (PSSM profile, PSSM information content and PSSM conservation score). The PSSM information of each individual protein was downloaded from 3D CONS, by querying the unbound PDB structure of each complex's partners. 3D CONS PSSM matrices are computed using the iterative BLAST algorithm (PSIBLAST) on each chain of a PDB file. By providing a reference structure of the complex, i.e. an experimentally solved bound conformation, DeepRank-GNN automatically computes the fraction of native contacts (*f*_nat_) that we used as the target value). As compared to the interface RMSD (iRMSD) or ligand RMSD (lRMSD) values, the *f*_nat_ value is capped between 0 and 1, thus giving the same weight to all bad quality models. For instance, two models very distant from the reference structure will be assigned a 0 *f*_nat_ value while they can be assigned very distinct iRMSD values (Supplementary Fig. S2), which can uselessly influence the network parameters optimization. In addition, the *f*_nat_ is less sensitive to the local motion at the interface of two proteins than the RMSD and is therefore more adapted to evaluate the quality of an interface.

#### 2.2.4 Network

We introduce here a GNN architecture, dubbed graph interaction network (GINet), whose general structure is represented in Figure 1B and detailed in Supplementary Information. As seen in this figure GINets are composed of a succession of graph convolution layers (GCL), non-linear activation (here ReLU) and pooling layers. Two distinct GCLs are applied at each convolution step. One GCL is applied on interface graphs, i.e. graphs with edges connecting nodes from distinct proteins, and a distinct GCL is used on internal graphs, i.e. graphs with edges connecting nodes from the same protein. The rationale behind this architecture is to extract information not only on the interaction itself but also on the propensity of each individual interface to establish an interaction.

#### 2.2.5 Training

The network was trained over 20 epochs on batches of 128 shuffled graphs. We used the mean square error loss (MSE loss) function using the *f*_nat_ values as the ground truth and the Adam algorithm (Kingma and Ba, 2017) with a learning rate of 0.001 to minimize the loss. A complete epoch (23 500 3D models) required 2.4 ± 0.9 h on 1 GPU (GeForce GTX 1080 Ti).

#### 2.2.6 Metrics computation

The area under the ROC curve (AUC), the hit rate and the success rate are computed to evaluate the performance of the scoring functions. To meet the requirement of these metrics that evaluate the discriminating ability of a binary classifier, we binarized the *f*_nat_ data using a 0.3 threshold: docking models with a *f*_nat_≥0.3 are considered to be of acceptable quality, while those with a *f*_nat_ < 0.3 are considered non-acceptable. This threshold differs from the CAPRI standard *f*_nat_ ‘acceptable’ quality threshold of 0.1 that is combined with additional iRMSD and lRMSD criteria (Lensink *et al.*, 2007). Herein, since no RMSD values are considered, we raised the acceptance threshold to the equivalent of the CAPRI standard *f*_nat_ ‘medium’ quality threshold of 0.3 to avoid misclassifying poor quality models (Supplementary Fig. S1).

The ROC curve is defined as the fraction of true positive rate (TPR) as a function of the fraction false positive rate while navigating through the ranking provided by the scoring function. The AUC is the integral of the ROC curve and is equal to 1 for an ideal classifier and 0.5 for a random classifier. The hitrate is defined as the percentage of hits retrieved within the top *N* ranks. The success rate is the number of complexes for which at least one acceptable quality model is retrieved within the top *N*.

### 2.3 Application 2

#### 2.3.1 MANY/DC benchmark

The MANY (Baskaran *et al.*, 2014) and the DC (Duarte *et al.*, 2012) datasets contain biological and crystal dimers, in balanced proportions (∼50%/50%), the latter being the consequence of crystal packing. The crystal dimers are indistinguishable from the biologic ones without a consistent knowledge of the complex. While the surface of biological interfaces is often larger than those of reported crystal dimers in different datasets, the DC dataset has been tuned to include biological and crystal dimers of comparable interface area. Herein, we used 80% of the MANY dataset (4591 dimers) to train our model and 20% (1148 dimers) to evaluate it. The retained model, i.e. the one displaying the minimum loss on the training set, was further tested on the DC dataset (161 dimers). All datasets are available from the SBGrid data repository https://data.sbgrid.org/dataset/843/.

#### 2.3.2 Graph generation and network architecture

The PPI interfaces were converted into residue graphs and each node was assigned PSSM information only (i.e. 20 features per node). The PSSM matrices from the DeepRank paper (Renaud *et al.*, 2021) were used. The exact same network architecture as described in Application 1 was used.

#### 2.3.3 Training

The network was trained over 50 epochs on batches of 128 shuffled graphs. We used the cross-entropy loss function using the biological (1)/crystal (0) annotations as the ground truth and the Adam algorithm (Kingma and Ba, 2017) with a learning rate of 0.001 to minimize the loss. A complete epoch (5739 3D models) required 1 min on 1 GPU (GeForce GTX 1080 Ti).

#### 2.3.4 Metrics computation

For this binary classification problem, we computed the accuracy, the specificity, the sensitivity and the precision to evaluate the performance of the DeepRank-GNN model.

## 3 Results

### 3.1 Application 1—the scoring of docking models

Docking is an *in silico* modeling approach commonly used to predict the 3D structure of biomolecular complexes. Docking involves two steps: The sampling, i.e. the exploration of the conformational interaction space to generate 3D models, and the scoring that aims to identify near-native models out of the pool of generated docking models. As illustrated by the Critical Assessment of PRedicted Interactions (CAPRI) initiative that frequently proposes blind predictions of experimentally determined 3D structures of protein complexes, there is still room for scoring functions improvement (Lensink *et al.*, 2016, 2021). Most scoring functions can be classified into physical energy-based, statistical potential-based and machine learning-based functions. They are constantly explored for improvement to either propose system-specific or broad-spectra scoring tools, which in some cases are also used to predict changes in binding affinities (Geng *et al.*, 2019).

Here, we demonstrate the use of DeepRank-GNN to score docking models of various complexes generated with a variety of docking software.

#### 3.1.1 Performance of 10-fold cross-validation on the BM5

**Fig. 2.**
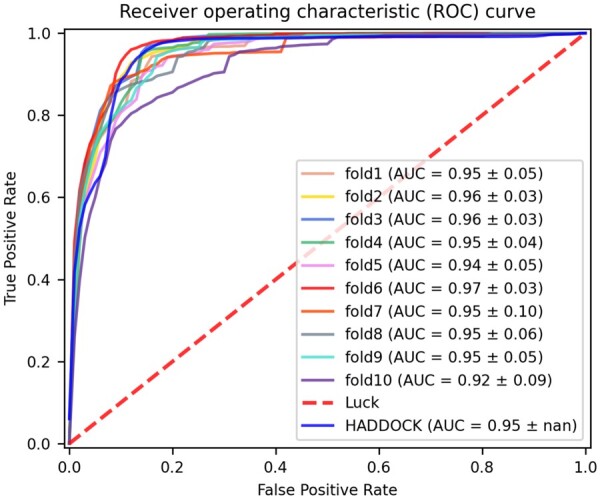
Comparison of DeepRank-GNN with HADDOCK scoring function on the BM5 set. Average ROC curves obtained with the models retained for each DeepRank-GNN fold, for the model trained on the full training set and HADDOCK score. A true positive case corresponds to a complex with *f*_nat_ > 0.3 correctly predicted. The number of true positive rate values is averaged over the number of complexes in the test dataset. The dashed line represents a random classifier

Ten-fold cross-validation was performed to analyze the performance and robustness of DeepRank-GNN on the task of scoring docking models. For each fold, we trained our model on the BM5 data set using 258 060 docking structures from 102 distinct complexes (see Section 2). The trained GINet models were validated on 63 250 docking structures from 25 complexes (see Supplementary Fig. S3). For each fold, we retained the generated model that minimizes the most the loss value on the evaluation set and evaluated its performance on the BM5 test set that consists of 375 700 docking structures from 15 complexes (described in Supplementary Table S1). As shown in Fig. 2, when defining positive docking models as those associated to a *f_nat_* ≥ 0.3 and averaging the TPR over the number of BM5 test complexes, most GINet models globally perform equally or better than the HADDOCK scoring function for 8 out of 10 DeepRank-GNN models, yielding an AUC ≥ 0.95 on the test set. We however notice a variation in the performance depending on the data subset used for the training and the evaluation of the models, which is particularly clear when we consider not only the complex-averaged TPR but also the standard deviation (Supplementary Table S3 and Supplementary Fig. S5) and the hit rates obtained on individual complexes (Supplementary Fig. S6), highlighting the dataset dependency of DeepRank-GNN performance. Among all DeepRank-GNN models, the highest performance is reached with the one generated in the fold6 (AUC = 0.97 ± 0.03). When training on all data (i.e. data from all folds), an AUC of 0.94 ± 0.06 is obtained. This last model was used to further assess the performances of DeepRank-GNN. To ease the comparison with other software, we provide a similar analysis using the standard CAPRI acceptable threshold instead of the *f*_nat_ in Supplementary Figure S4.

#### 3.1.2 Rank correlation

Since rank correlation is a good indicator of the predictiveness of a score, we computed the Spearman ρ correlation between the *f*_nat_ and the DeepRank-GNN scores obtained with the GINet model trained on the full dataset on the entire test set. We observe an average Spearman ρ correlation of 0.49 ± 0.14, the highest correlation being obtained for 1PPE (ρ = 0.63), the lowest for 2OZA (ρ=0.14) (Supplementary Fig. S7). Interestingly, 1PPE constitutes an *easy* case with 11.5% of good docking models (*f*_nat_≥0.3) generated, while 2OZA constitutes a more difficult case with 4.7%. Overall, we observe a good ability to identify near-native models in the top-ranked model with impressive success rates of 66.7% (7, 12 and 14 over 15 test complexes) at top1, top5 and top10 and 73.3% at top50.

The performance considerably increases when considering only the HADDOCK refined models (i.e. it1 and itw models as defined in Supplementary Information) of the test set with an average Spearman correlation of 0.69 ± 0.23, the highest correlation being obtained for 1PPE (ρ = 0.89), the lowest for 1F6M (ρ=0.02) (Supplementary Fig. S8). Here again, 1F6M constitutes a difficult case with 102 good models in the entire pool of docking models, 100 of them consisting in refined structure of the bound complex and representing very low diversity in the binding mode. The success rate is similar to the one on the entire set with 66.7% (10 over 15 test complexes) at top1, top5 and top10 and 93.3 at top50. In both scenarios, success rates of 46.7, 66.7 and 80% (7, 10 and 12 over 15) are obtained with HADDOCK scores for the top1, top5 and top10, respectively (Table 2). For convenience, a similar analysis using the classic CAPRI labels is provided in Supplementary Figure S9.

**Table 2.** AUC and success rates of HADDOCK and DeepRank-GNN (trained on the full training set) on the BM5 test dataset when considering HADDOCK refined models only

	AUC	Success rates (%)			
		Top 1	Top 5	Top 10	Top50
DeepRank-GNN	0.85 ± 0.16	**66.7**	66.7	66.7	**93.3**
HADDOCK	0.85	46.7	66.7	**80**	86.7

The bold values indicate the best value for each column.

#### 3.1.3 Comparison to external software on the CAPRI score test set

We evaluated the performance of DeepRank-GNN, DeepRank, DOVE, HADDOCK and iScore on the CAPRI score set. DeepRank and DOVE are two CNN-based scoring approaches, iScore is graph-kernel based, and HADDOCK uses a classic scoring function that consists of a linear combination of energy terms. The CAPRI score set consists of 13 complexes for which 497 to 1987 models have been generated by different groups using a wide diversity of docking tools and protocols (Lensink and Wodak, 2014). When considering the AUC DeepRank-GNN stands on top together with iScore with an average AUC of 0.71 and 0.64, respectively (Table 3). However, in terms of early enrichments (success rate of top *N* models for N ≤ 5), iScore scores best followed by HADDOCK and GNN-DOVE (Table 3). Note that among these three tools, iScore and GNN-DOVE are using graph representations.

**Table 3.** Comparison of the performance obtained on the CAPRI Scoreset

	AUC	Success rates (%)			
		Top 1	Top 2	Top 5	Top100
iScore	0.64 ± 0.29	**38.5**	**46.2**	46.2	**76.9**
DOVE	0.48 ± 0.22	7.7	7.7	15.4	**76.9**
GNN-DOVE	0.54 ± 0.23	15.4	30.8	**53.8**	**76.9**
DeepRank	0.59 ± 0.28	15.4	15.4	15.4	69.2
DeepRank-GNN	**0.71 ± 0.24**	7.7	23.1	38.5	**76.9**
HADDOCK	0.55 ± 0.27	23.1	23.1	23.1	69.2

*Note*: A true positive case corresponds to a complex with *f*_nat_ > 0.3 correctly predicted. To ease the comparison with other software we provide a figure using the standard CAPRI acceptable threshold instead of the *f*_nat_ in Supplementary Figure S9.

#### 3.1.4 Computational performance

The graph representation of the interface not only provides a natural way of representing PPI interfaces, but it also considerably improves the computation performance in terms of storage, data generation and learning speed as compared to the use of grids and CNN. To quantify it, we compared the graph generation step of DeepRank-GNN to the grid generation step of DeepRank on the CAPRI score set (Supplementary Tables S4–S6) as well as each protocol’s training speed (Supplementary Table S7) using MPI distributed processes on 4 CPUs. The results show that the graph generation is on average 20 times faster than the 3D grid generation in CNN (0.65 ± 0.31 versus 12.4 ± 3.3 second per model) and requires ∼22 times less storage space (0.14 ± 0.1 versus 3.07 ± 0.4 MB per model). It is worth noting that default settings were used for each approach and that DeepRank computes additional atomic-level descriptors leading to a total of 72 descriptors against 48 in DeepRank-GNN (Supplementary Table S8). To fairly compare the computation performance in the training phase, we retained comparable residue-level features for the two protocols corresponding to 48 and 58 features for DeepRank-GNN and DeepRank respectively, and split the CAPRI score set into 80% training and 20% evaluation sets. We trained the GINet model of DeepRank-GNN described in section 3.1 and the default 3D-CNN implemented in DeepRank over 10 epochs. The results show a remarkable speed difference, DeepRank-GNN being ∼25 times faster than DeepRank when no data augmentation is used in the latter (Supplementary Table S7).

### 3.2. Application 2—biological versus crystal interfaces classification

X-ray crystallography is, as per 2022, the most used experimental method to solve the 3D structure of proteins. Despite most X-ray structures being reliable and of high quality, it is not rare to obtain erroneous structures. Among the common errors are the incorrect residues fitting into the electron density maps and the observation of artificial oligomers due to crystal packing. The latter can lead to dramatically wrong conclusions and mislead researchers in their study. It is therefore essential to provide tools to annotate crystallographic dimers as reliable (i.e. biological) or not (crystal).

In this section, we evaluate the performance of DeepRank-GNN in discriminating biological and crystal interfaces from the DC dataset.

#### 3.2.1 DeepRank-GNN classification performances

Deeprank-GNN was trained and evaluated on 5739 dimers from the MANY dataset, with 80% of the dimers constituting the training set and 20% the validation set. The network was trained over 50 epochs and we retained the model minimizing the loss on the evaluation set. This model was further tested on the DC dataset that contains 80 biological and 81 crystal interfaces with comparable interface areas. We observe interesting performance with an accuracy of 82%, a specificity of 81%, a sensitivity of 83% and a precision of 82%. Note that 11 structures overlap between the DC and the MANY datasets and that removing them only slightly affects the performances (accuracy: 81%, specificity: 82%, sensitivity: 79.2%, precision: 80.3%).

#### 3.2.2 Comparison to external software

We recently evaluated and published the performance of the non-commercial software PISA, PRODIGY-crystal and DeepRank on the DC dataset. The DeepRank-GNN model that we present here ranks second in terms of accuracy (82%) behind DeepRank (86%) and ahead of PISA (79%) and PRODIGY-CRYSTAL (74%) (Table 4).

**Table 4.** Comparison of the accuracy obtained on the biological versus crystal interfaces classification task

	PISA	PRODIGY-Crystal	DeepRank	DeepRank-GNN
Accuracy (%)	79	74	**86**	82

## 4 Conclusion

We have developed DeepRank-GNN, a new computational framework to learn and predict interaction patterns from protein–protein interfaces. DeepRank-GNN is provided as a freely accessible python3 package (https://github.com/DeepRank/DeepRank-GNN). The framework encompasses pre-processing tools that take PPI PDB files as input, converts the interface of interaction into residue-level graphs and automatically assigns biologically relevant features to the graphs. In the second step, the graphs can be used to train, evaluate and test a provided or user-defined GNN to make problem-specific predictions. DeepRank-GNN has been designed to be applied to various PPI-related projects and offers the users many options such as the possibility to select features, to use any type of target values, to reweight the scoring functions for classification tasks, to design their own GNN architecture etc.

As a demonstration, we applied DeepRank-GNN to the task of scoring docking models of various complexes from the Docking Benchmark 5 (BM5) and the CAPRI score set. The trained models (one per fold) globally display a data dependency, yet most of them compete with- or outperform the HADDOCK scoring function. The scoring performance of a model trained on the entire training set was further evaluated on the independent CAPRI score set and compared to HADDOCK, DeepRank, DOVE, GNN-DOVE and iScore. DeepRank-GNN and iScore rank 1st and 2nd in this task in terms of AUC, iSCore showing better early enrichment. Interestingly, among all scoring functions mentioned in this work, DeepRank-GNN is the only one that does not contain energy terms, highlighting that simple geometric and physico-chemical properties could be as informative as (or more than) the approximative energy terms used in most scoring functions. Further optimization of the hyperparameters of the GINet or the design and application of new GNNs could help improve the performance observed here.

We also trained a DeepRank-GNN network to discriminate biological interfaces from crystal interfaces. In this task, we obtained an accuracy of 82%, competitive with the state-of-the-art methods DeepRank (86%), PISA (79%) and PRODIGY-crystal (74%). The graph generation plus the DeepRank-GNN model training, evaluation and test were performed in less than 2 h.

Overall, DeepRank-GNN is a versatile software that should help the community learn patterns from protein–protein interfaces in a time-efficient manner. We present a single GNN architecture in this article that can be reused as such or derived and replaced. The DeepRank framework offers many perspectives of extension such as the application to single proteins (e.g. for genetic variant pathogenicity prediction), to larger multimeric states (e.g. for larger complex quality prediction), or the integration of other GNN architectures such as the E(n)-equivariant graph neural networks to run molecular dynamics simulations.

## Supplementary Material

btac759_Supplementary_Data

## Data Availability

The BM5 and CAPRI score set docking models are obtained from the DeepRank paper (Renaud *et al.*, 2021) and can be downloaded from: https://data.sbgrid.org/dataset/843/.

## Software availability

The software is available on Github (https://github.com/DeepRank/Deeprank-GNN) and the documentation is available online (https://deeprank-gnn.readthedocs.io/). The models mentioned in the article, the content of the BM5 dataset, and the target and the predicted values for the BM5, CAPRI and DC test set are provided on GitHub (https://github.com/DeepRank/Deeprank-GNN/tree/master/paper_pretrained_models).
