# Supplementary material for: DeepRank-GNN: a graph neural network framework to learn patterns in protein–protein interfaces
Source: Bioinformatics. 2022 Nov 24;39(1):btac759. doi: 10.1093/bioinformatics/btac759 (PMC9805592; doi:10.1093/bioinformatics/btac759)
Supplement: btac759_Supplementary_Data [file btac759_supplementary_data.pdf]

DeepRank-GNN: A Graph Neural Network Framework to Learn Patterns in Protein-Protein Interfaces

Manon Réau[1,#], Nicolas Renaud[2,#] , Li C. Xue[3], Alexandre M. J. J. Bonvin[1,*]

[1]Computational Structural Biology Group, Department of Chemistry, Bijvoet Centre, Faculty of Science, Utrecht University, Utrecht, 3584CH, The Netherlands., [2]Netherlands eScience Center, Science Park 140, 1098 XG, Amsterdam, The Netherlands., [3]Center for Molecular and Biomolecular Informatics, Radboudumc, Greet Grooteplein 26-28, 6525 GA Nijmegen, The Netherlands

*To whom correspondence should be addressed.

# These authors contributed equally.

Table of contents

Generation of docking models with HADDOCK 2

Automated weights computation for CrossEntropy Loss function 2

Graph Interaction Network (GINet) 3

Tables and Figures

Figure S1 *DeepRank-GNN diagram. The graphs generation module of DeepRank-GNN requires PDB files, features and target specifications as input data. The target values can be either provided by the user or automatically computed in a docking benchmark scenario upon provision of reference structures. Generated graphs are stored in HDF5 format for memory and I/O optimization. Graphs are fed to the training module that additionally requires a defined GNN architecture and hyperparameters. The user can save all generated models or save only the last one, intermediate models, or the best one based on the loss value on the evaluation set. The trained model(s) can be further applied to test sets and unlabelled graphs for independent validation and prediction.* 4

Table S1 *CAPRI quality criteria automatically computed by DeepRank-GNN upon provision of a reference structure* 4

Table S2*: Composition of the test set. Note that complexes displaying important clashes could not be converted into graphs.* 5

Figure S2 *(A) Correlation between iRMSD and fnat values, (B) superimposition of a docking model (green and cyan) associated to a fnat > 0.6 and an iRMSD > 6A on the reference structure (1JTG - grey).* 5

Figure S3 *Evolution of the loss value obtained on the training and the evaluation sets over 20 epochs. Results are shown for the 10 -folds of the cross validation considering PSSM information or not.* 6

Figure S4 Comparison of DeepRank-GNN with HADDOCK scoring function on the BM5 set. *(A) Average ROC curves obtained with the models retained for each DeepRank-GNN fold and HADDOCK score. A true positive case corresponds to a complex with an acceptable (or better) CAPRI class correctly predicted. The number of True Positive Rate value is averaged over the number of complexes in the test dataset. The dashed line represents a random classifier. (B) Success rates of HADDOCK and DeepnRank-GNN on the BM5 test dataset. An acceptable CAPRI class correspond to fnat ≥ 0.1 OR (i-RMSD ≤4 A AND l-RMSD ≤ 10 A)* 7

Table S3: *Performance of the best model obtained per fold on the BM5 test dataset.* 7

Figure S5*: Average Receiver operating characteristic curves (ROC) obtained with the models retained for each DeepRank-GNN fold and HADDOCK score on the BM5 test dataset. A true positive case corresponds to a complex with fnat ≥ 0.3 correctly predicted. The number of True Positive Rate value is averaged over the number of complexes in the test dataset. The dashed line represents a random classifier* 8

Figure S6: *Hitrate obtained with the models retained for each DeepRank-GNN fold and HADDOCK score on each complex from the BM5 test set. A true positive case corresponds to a complex with fnat ≥ 0.3 correctly predicted.* 10

Figure S7: *Correlation plots of the measured fnat (target) and the DeepRank-GNN score (prediction) on the BM5 test dataset (it0/it1/itw). The color code provides indications in the number of models associated to a plot area. The Spearman (rank) correlation is provide for each complex.* 13

Figure S8*: Correlation plots of the measured fnat (target) and the DeepRank-GNN score (prediction) on the BM5 test dataset (it1 and itw). The color code provides indications in the number of models associated to a plot area. The Spearman (rank) correlation is provide for each complex.* 14

Figure S9 *Comparison of the performance obtained on the CAPRI Scoreset. See legend of Fig.S4.* 15

Table S4*: Performance of the* ***graph*** *generation step of* ***DeepRank-GNN*** *on the 13 complexes (16666models) of the CAPRI score set using MPI distributed processes (4 CPUs).* 16

Table S5*: Performance of the* ***grids*** *generation step of* ***DeepRank*** *on the 13 complexes (16666models) of the CAPRI score set using MPI distributed processes (4 CPUs) with no rotation of the input model.* 17

Table S6*: Performance of the* ***grids*** *generation step of* ***DeepRank*** *on the 13 complexes (16666models) of the CAPRI score set using MPI distributed processes (4 CPUs) with 5 rotation of the input model, i.e. 6 orientation per model in total.* 18

Table S7: *Comparison of the computational performance of DeepRank-GNN and DeepRank in the training/evaluation phase using MPI distributed processes (4 CPUs). 80% of the 16666 CAPRI models fall into the training set, 20% into the evaluation set.* 18

Table S8: *DeepRank and DeepRank-GNN default features. The residue-level features highlighted in bold characters have been considered to train DeepRank and DeepRank-GNN in the comparative study detailed in section 3.3* 19

Generation of docking models with HADDOCK

Docking with HADDOCK follows a three step process: 1) in the first stage (it0), the docking is performed from the separated and randomly rotated starting conformations, treating the proteins as rigid units,; 2) in the second stage (it1), a semi-flexible refinement is performed, consisting of a simulated annealing in torsional space (with fixed bond lengths and angles) introducing flexibility first along the side chains at the interface (i.e. those within 5 Å of the partner protein), and second in both backbone and side chains of the interface residues; 3) in the last stage (itw), the models are subjected to a final energy minimization (and/or short refinement in explicit solvent (water)). Each HADDOCK step has its own scoring function and restraints can be provided to guide the docking with a priori information.

- $HADDOCK_{it0} = 0.01 \times E_{vdw} + 1.0 \times E_{elec} + 1.0 \times E_{desolv} - 0.01 \times BSA$
- $HADDOCK_{it1} = 1.0 \times E_{vdw} + 1.0 \times E_{elec} + 1.0 \times E_{desolv} - 0.01 \times BSA$
- $HADDOCK_{itw} = 1.0 \times E_{vdw} + 0.2 \times E_{elec} + 1.0 \times E_{desolv}$

In order to ensure a suitable number of near-native models (i.e. iRMSD to the reference structure ≤ 4 Å), we combined models from 5 docking scenarios with increasing level of a priori information: 1) docking with random surface patch restraints (10000/400/400 models for it0/it1/water stages), 2) docking with center of mass restrains (10000/400/400 models for it0/it1/water stages), 3) docking with true interface residues defined within a 5 A distance to the partner protein (1000/400/400 for it0/it1/water stages), 4) docking with true interface residues defined within a 3.9 A distance to the partner protein (1000/400/400 for it0/it1/water stages) and 5) refinement of the bound complex (50/50 it1/water stages).

Automated weights computation for CrossEntropy Loss function

The cross-entropy loss function is commonly used for classification tasks and more especially when dealing with multiple classes. The cross-entropy loss function computes the probability of each class *y*, and applies a logarithmic penalty based on how far is the prediction from the ground truth value.

For a binary classification tasks, the cross-entropy loss function is defined as:

$$Loss_{CE} = -\sum_{i=1}^{2} \quad y_i \, log \, log \, (p_i) \; = \; -(y \, log \, log \, (p) \; + (1 - y) \, log \, log \, (1 - p) \,)$$

Where *i* spans the number of classes, y_i is the ground truth value (0 or 1), and p_i is the probability of the class *i*, and where $p_1 = 1 - \; p_2$. Provided that $y_n$is the target (i.e. the correct class to predict), then $y_n = 1$ and all other $y_i = 0$. We can thus simplify the equation for multi-classes tasks to:

$$Loss_{CE} = - \, log \, y_n log \, (p_n)$$

$p_n$being the probability of the target class y_n and being computed with the SoftMax activation function:

$$Loss_{CE} = -\log y_n log\left(\frac{exp\,(x_{n,y_n})}{\sum_{c=1}^{C}\quad exp\,(x_{n,c})}\right)$$

where x_{n,y_n} is the predicted value for the target class, *c* spans the number of classes, and $x_{n,c}$ is the predicted value for each class *c*.

In PyTorch, the cross-entropy loss is computed for each entry n of the input batch as follow:

$$Loss_{CE} = \{l_1, l_2, \ldots, l_N\};$$

With

$$l_n = -\log w_{y_n} y_n log\left(\frac{exp\,(x_{n,y_n})}{\sum_{c=1}^{C}\quad exp\,(x_{n,c})}\right) = -\log w_{y_n} log\left(\frac{exp\,(x_{n,y_n})}{\sum_{c=1}^{C}\quad exp\,(x_{n,c})}\right) = -w_{y_n}\log\log(p_n)$$

w_{y_n} being the weight, or scaling factor, assigned to the target class. By default, w_{y_n} is set to 1 for each class c, meaning that all classes equally contribute to the loss calculation. The batch loss can be computed as the mean (default in pytorch and DeepRank-GNN) or the sum of individual losses. However, in case of unbalanced training dataset, it is recommended to weight the different classes with higher weights for the minority class(es) to avoid optimizing the neural network weights solely based on majority classes. An agreed upon technique to weight the contribution of each classes in the loss function is to assign weights that are inversely proportional to the frequency of each class in the training set.

In DeepRank-GNN, the users can input their own weights or let DeepRank-GNN automatically compute them as follow:

- Compute the frequency of each class
- Convert it into a percentage

Example:
We have a training dataset of 400 graphs, 4 classes (0,1,2,3) split as follow:

- Class 0: 200 graphs
- Class 1: 50 graphs
- Class 2: 50 graphs
- Class 3: 100 graphs

The frequency F is given by:

$$F = \left[\frac{1}{200}, \frac{1}{50}, \frac{1}{50}, \frac{1}{100}\right] = [0.005, 0.02, 0.02, 0.01]$$

It is then transformed into a frequency percentage:

$$weights = \left[\frac{0.005}{\sum_{i=0}^{3}\quad F_i}, \frac{0.02}{\sum_{i=0}^{3}\quad F_i}, \frac{0.02}{\sum_{i=0}^{3}\quad F_i}, \frac{0.01}{\sum_{i=0}^{3}\quad F_i}\right] = [0.0909, 0.3636, 0.3636, 0.1818]$$

Graph Interaction Network (GINet)

The convolution layers in the GINet are inspired by the graph attention network (GAT) described by Veličković et al., 2018 and the edge aggregated graph attention network (EGAT) from Mahbub and Bayzid, 2020. Attention mechanism (Veličković et al., 2018) is used to weight the contribution of individual neighbors in the new state of a node. When applied to PPIs, we expect attention mechanism to learn favorable or deleterious contacts in a local environment.

The feature representation of node i, $h_i \in R^{f_i}$, is transformed into a new feature representation $h'_i \in R^{f_o}$ (where f_i and f_o are respectively the number of input and output features) through the aggregation of the feature representation of neighbouring nodes N_i, i.e. the first ordered neighbours of i, including node i itself. We use the weighted sum of the neighbours feature representation as an aggregator,

$$\overrightarrow{h'}_i = \sum_{j \in Ni} \quad \alpha_{ij} W^f \overrightarrow{h_j} \#(1)$$

where $W^f \in R^{f_o \times f_i}$ is the learnable parameters used in the linear transformation of the node features representation and where the weights α_{ij} are learned through an attention mechanism described in equations 2 and 3. Each edge of the graph is assigned an attention score s_{ij} given by

$$s_{ij} = Leak\_ReLU\left(\vec{a}^T\left(\left[W^f\overrightarrow{h_i}\|W^f\overrightarrow{h_j}\|W^e e_{ij}\right]\right)\right) \#(2)$$

Where e_{ij} is edge feature, which herein corresponds to the interaction strengh as explained in the Featurization section of the main manuscript, $W^e \in R^{f_e \times f_e}$ is the learnable parameter used in the linear transformation of the edge feature representation (f_e being the number of edge features), and $\vec{a}^T \in R^{2\times f_o + f_e}$ is the attention mechanism. || denotes vector concatenation. s_{ij} is further normalized by a SoftMax activation function to give a probability distribution α_i (between 0 and 1) over the node neighbours N_i, with α_{ij} reflecting how much the neighbouring node j should contribute to the new representation of node i.

$$\alpha_{ij} = softmax\left(s_{ij}\right) = \frac{exp\ exp\left(s_{ij}\right)}{\sum_{k\in Ni} \quad exp\ exp\left(s_{ik}\right)} \#(3)$$

As described by Mahbub and Bayzid, 2020 and shown in equation 3, the calculation of the attention score considers the node features $h_i \in R^{f_i}$ and $h_j \in R^{f_i}$ as in the original GAT paper plus the edge feature $e_{ij} \in R^{f_o}$. The addition of the edge feature, which herein corresponds to the interaction strength, is essential for PPI interfaces studies as it is well established that the potential energy of a system depends on the atomic pairwise distances. In a coarse-grained system with residue-level representation, the same trend transfers to residue-residue distances.

Pooling layers are used to reduce the number of nodes in the graph, which depends on the input 3D model, and learn higher level features, i.e. features of group of nodes, during the training. The pooling operation is here performed on clusters of highly interacting nodes that can be computed using either a Markov Cluster Algorithm (MCL)(Enright *et al.*, 2002), or the Louvain community detection algorithm (Blondel *et al.*, 2008). Both clustering methods rely on the interaction strength and therefore cluster nodes that are close to each other in 3D space on the fly. A max pooling is then applied on these clusters. This pooling operation aggregates the nodes of a given cluster into a single node whose feature values are given by the maximum feature values across the cluster. Interface and internal edges are then drawn to connect these new nodes. The edge features are here obtained by summing up all the edge features of the nodes belonging to the cluster.

Figure S1 *DeepRank-GNN diagram. The graphs generation module of DeepRank-GNN requires PDB files, features and target specifications as input data. The target values can be either provided by the user or automatically computed in a docking benchmark scenario upon provision of reference structures. Generated graphs are stored in HDF5 format for memory and I/O optimization. Graphs are fed to the training module that additionally requires a defined GNN architecture and hyperparameters. The user can save all generated models or save only the last one, intermediate models, or the best one based on the loss value on the evaluation set. The trained model(s) can be further applied to test sets and unlabelled graphs for independent validation and prediction.*

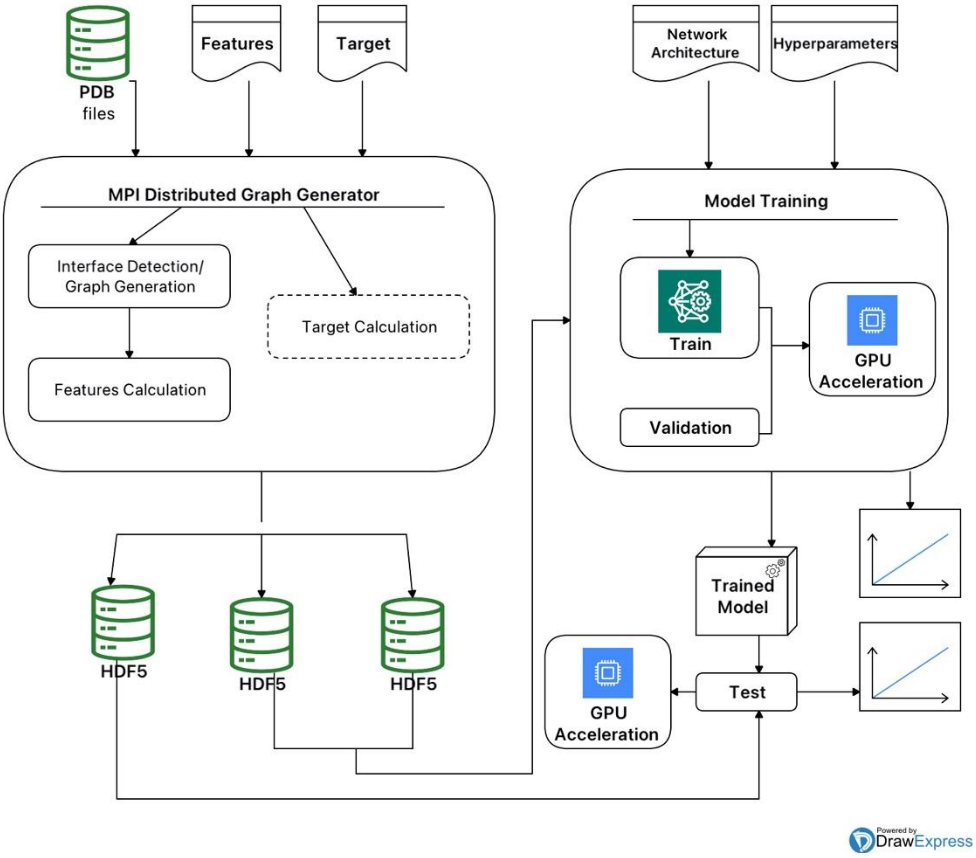

***Table S1** CAPRI quality criteria automatically computed by DeepRank-GNN upon provision of a reference structure*

CAPRI quality criteria	Definition
interface RMSD (iRMSD)	RMSD between superimposed interface residues
ligand-RMSD (lRMSD)	RMSD between chains B after superimposition of chains A
fraction of native contacts (f_{nat})	the fraction of reference interface contacts preserved in the interface of the docking model; the interface is defined as any pair of heavy atoms from two chains within 5Å of each other.
dockQ	(Basu and Wallner, 2016)
binary class	$0\colon irmsd \geq 4$ Å, $1\colon irmsd < 4$Å
capri irmsd classes	$1\colon irmsd < 1$Å, $2\colon irmsd < 2$Å, $3\colon irmsd < 4$Å, $4\colon irmsd < 6$Å, $0\colon irmsd \geq 6$ Å

***Table S2**: Composition of the test set. Note that complexes displaying important clashes could not be converted into graphs.*

	**fnat >= 0.3**	**fnat < 0.3**	**Total generated graphs**	**fraction good models**	**DeepRank-GNN scoring time per complex (seconds)**	**DeepRank-GNN scoring time per model (seconds)**
**1AK4**	2352	22948	25300	9,3 %	900,7	3,6E-02
**1BVN**	2337	22963	25300	9,2 %	861,0	3,4E-02
**1CGI**	751	24549	25300	3,0 %	946,0	3,7E-02
**1F6M**	102	25197	25299	0,4 %	874,8	3,5E-02
**1H1V**	211	11610	11821	1,8 %	228,5	1,9E-02
**1IBR**	100	25198	25298	0,4 %	970,5	3,8E-02
**1OPH**	100	25199	25299	0,4 %	886,0	3,5E-02
**1OYV**	1951	23349	25300	7,7 %	457,4	1,8E-02
**1PPE**	2913	22386	25299	11,5 %	467,3	1,8E-02
**1XQS**	2091	23209	25300	8,3 %	449,5	1,8E-02
**2OZA**	693	22807	23500	2,9 %	426,4	1,8E-02
**2SNI**	2488	22812	25300	9,8 %	465,7	1,8E-02
**2YVJ**	1215	24085	25300	4,8 %	704,9	2,8E-02
**2Z0E**	257	25043	25300	1,0 %	823,1	3,3E-02
**3K75**	3293	21986	25279	13,0 %	877,1	3,5E-02
			**average % of good models**	5,6 %	**average speed per model**	2,8E-02
			**standard deviation**	4,5 %		

Figure S2 *(A) Correlation between iRMSD and fnat values, (B) superimposition of a docking model (green and cyan) associated to a fnat > 0.6 and an iRMSD > 6A on the reference structure (1JTG - grey).*

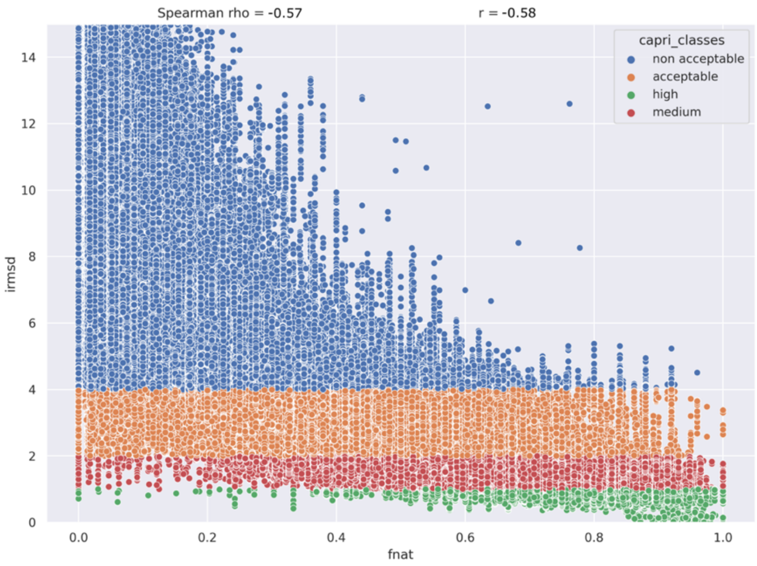

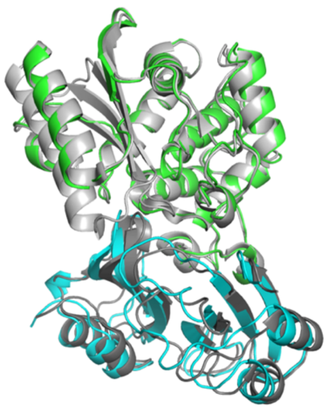

Figure S3 *Evolution of the loss value obtained on the training and the evaluation sets over 20 epochs. Results are shown for the 10 -folds of the cross validation considering PSSM information or not.*

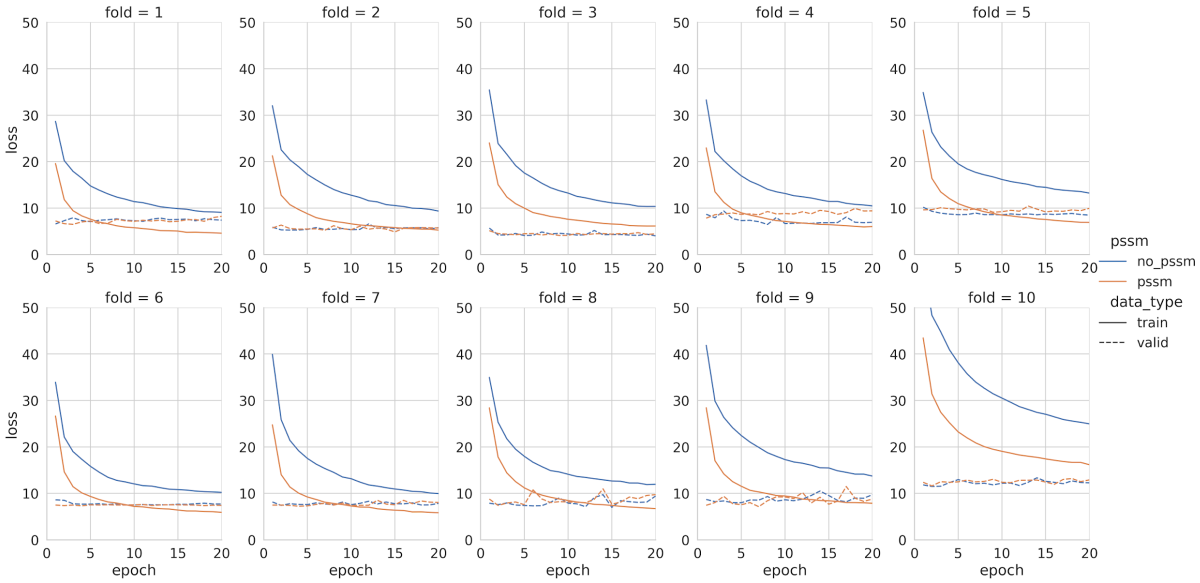

Figure S4 Comparison of DeepRank-GNN with HADDOCK scoring function on the BM5 set. *Average ROC curves obtained with the models retained for each DeepRank-GNN fold and HADDOCK score. A true positive case corresponds to a complex with an* acceptable *(or better) CAPRI class correctly predicted. The number of True Positive Rate values is averaged over the number of complexes in the test dataset. The dashed line represents a random classifier.*

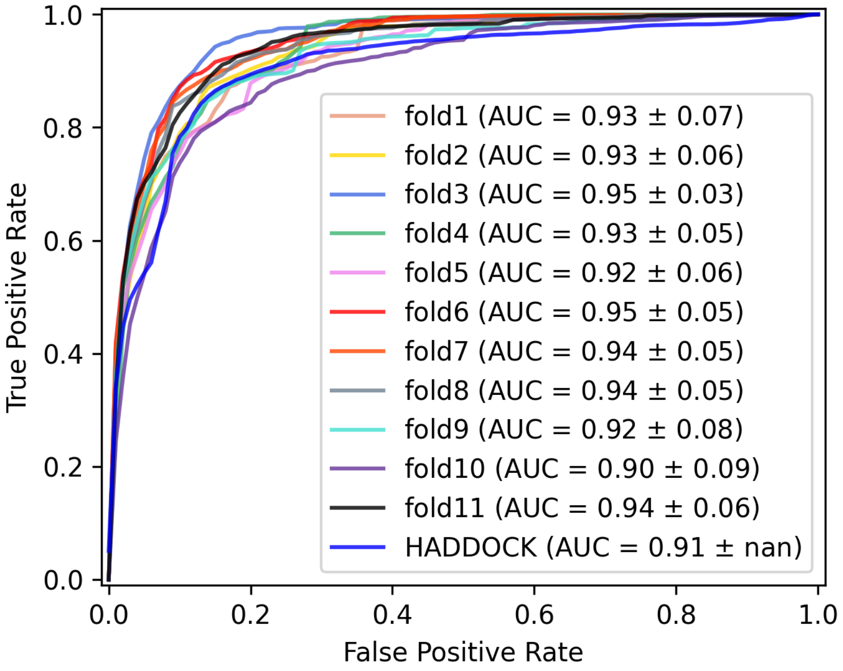

Table S3: *Performance of the best model obtained per fold on the BM5 test dataset with Deeprank-GNN.*

	**scoring performance**	**regression**		**classification** (acceptable quality: fnat >= 0.3)		
**fold**	**avg AUC**	**avg MSE**	**avg r2**	**avg accuracy**	**avg true positive rate**	**avg true negative rate**
**1**	0.95±0.05	0.02±0.01	-0.68±1.78	0.94±0.03	0.62±0.37	0.96±0.03
**2**	0.96±0.03	0.02±0.01	-0.17±1.01	0.94±0.03	0.49±0.35	0.97±0.03
**3**	0.96±0.03	0.02±0.01	-0.52±1.59	0.93±0.03	**0.63±0.34**	0.95±0.04
**4**	0.95±0.04	0.02±0.01	-1.53±3.11	0.91±0.05	0.75±0.27	0.93±0.06
**5**	0.94±0.05	0.02±0.02	-0.05±0.88	0.95±0.03	0.47±0.39	0.97±0.03
**6**	**0.97±0.03**	0.02±0.02	**0.15±0.51**	**0.95±0.03**	0.49±0.38	**0.98±0.03**
**7**	0.95±0.10	0.02±0.01	-0.43±1.54	0.94±0.03	0.62±0.37	0.96±0.04
**8**	0.95±0.06	**0.01±0.01**	-0.06±0.87	**0.95±0.03**	0.59±0.38	0.97±0.03
**9**	0.95±0.05	**0.01±0.01**	-0.26±1.71	**0.95±0.02**	0.52±0.33	0.97±0.03
**10**	0.92±0.09	0.02±0.02	-0.09±1.05	**0.95±0.04**	0.26±0.3	**0.98±0.03**

***Figure S5**: Average Receiver operating characteristic curves (ROC) obtained with the models retained for each DeepRank-GNN fold and HADDOCK score on the BM5 test dataset. A true positive case corresponds to a complex with fnat ≥ 0.3 correctly predicted. The number of True Positive Rate values is averaged over the number of complexes in the test dataset. The dashed line represents a random classifier*

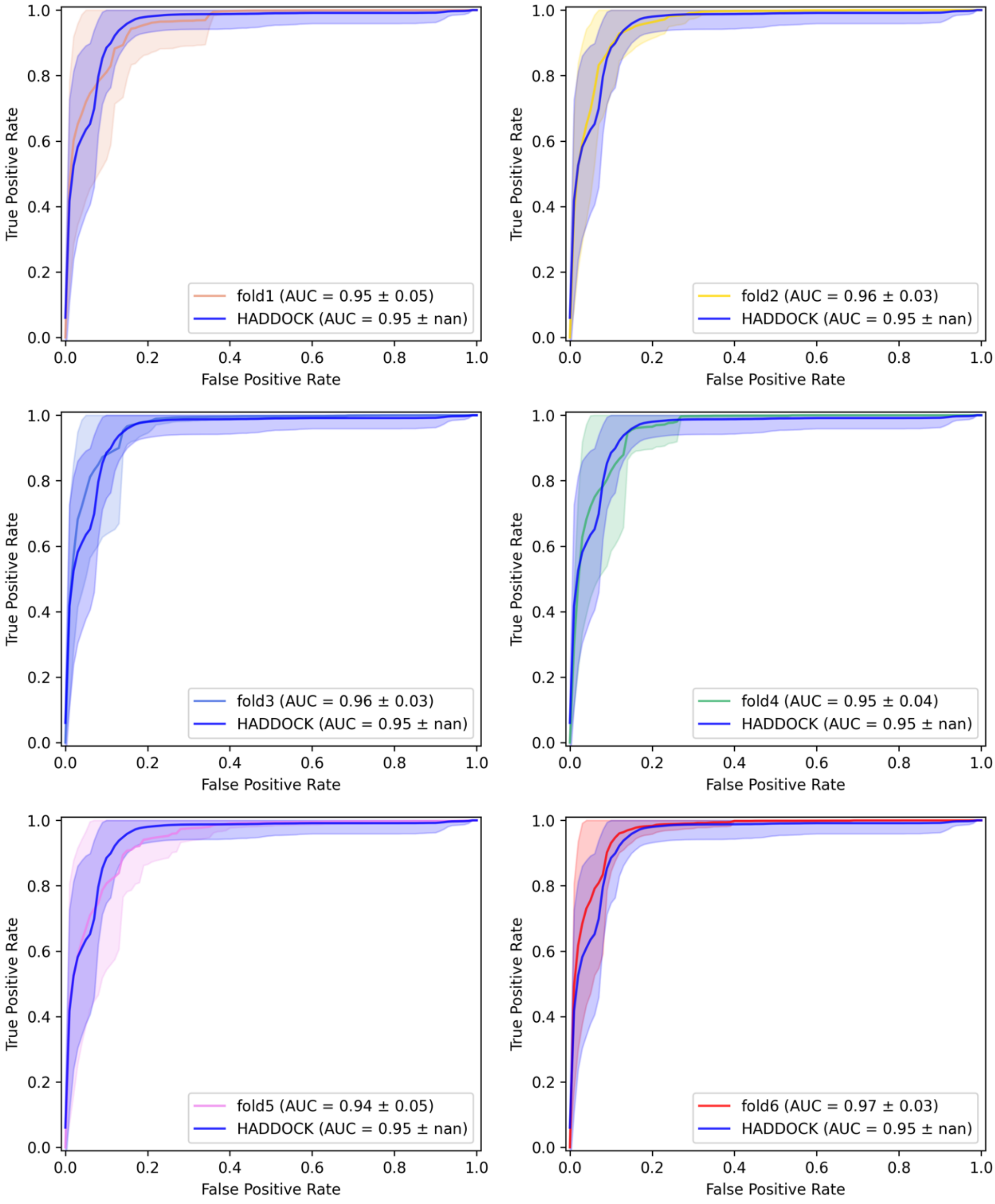

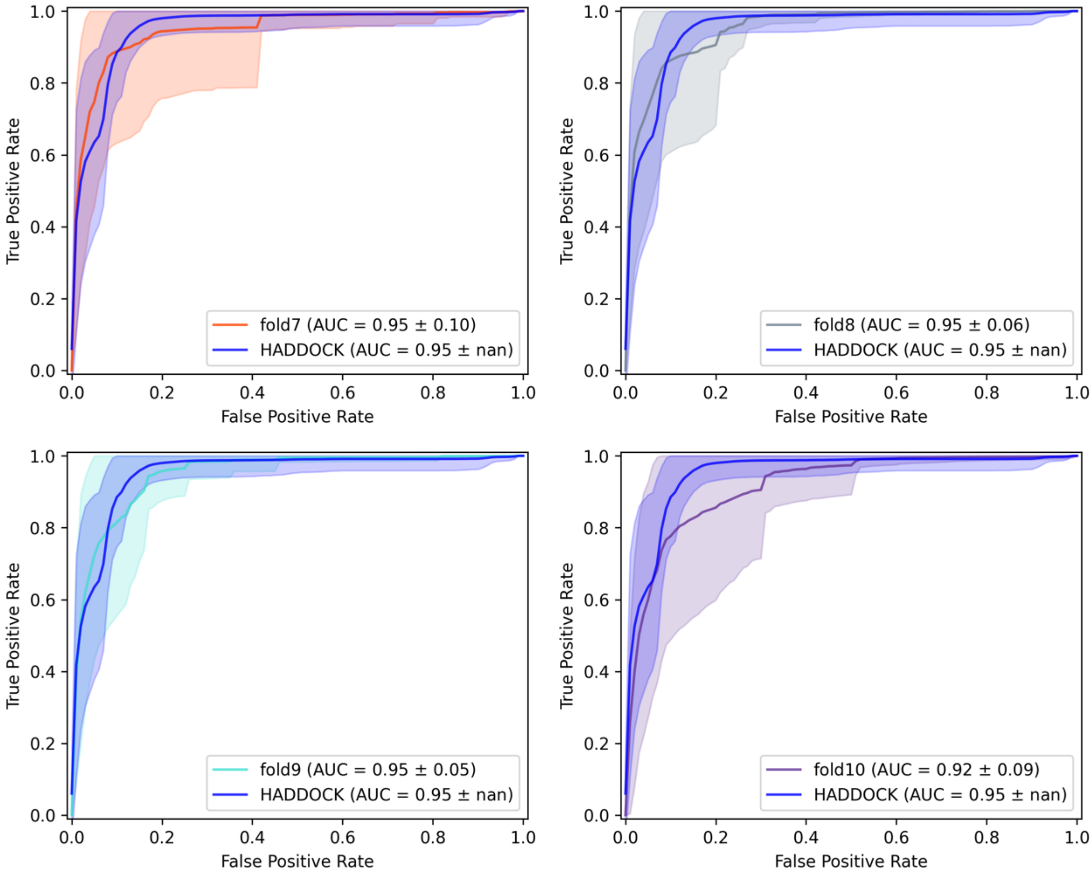
True Positive Rate
False Positive Rate
fold7 (AUC = 0.95 ± 0.10)
HADDOCK (AUC = 0.95 ± nan)
fold8 (AUC = 0.95 ± 0.06)
HADDOCK (AUC = 0.95 ± nan)
fold9 (AUC = 0.95 ± 0.05)
HADDOCK (AUC = 0.95 ± nan)
fold10 (AUC = 0.92 ± 0.09)
HADDOCK (AUC = 0.95 ± nan)

Figure S6: *Hitrate obtained with the models retained for each DeepRank-GNN fold and HADDOCK score on each complex from the BM5 test set. A true positive case corresponds to a complex with fnat ≥ 0.3 correctly predicted.*

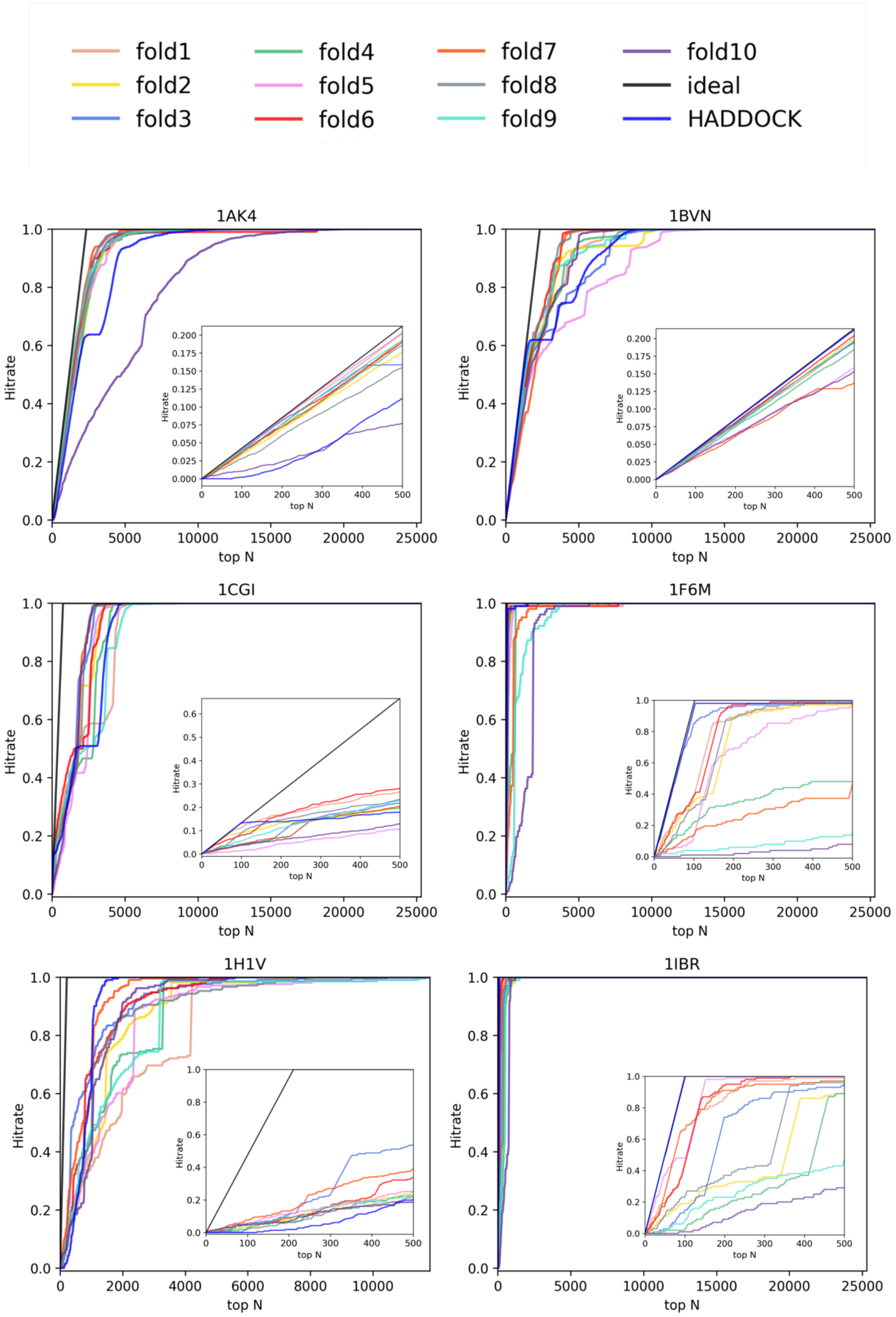

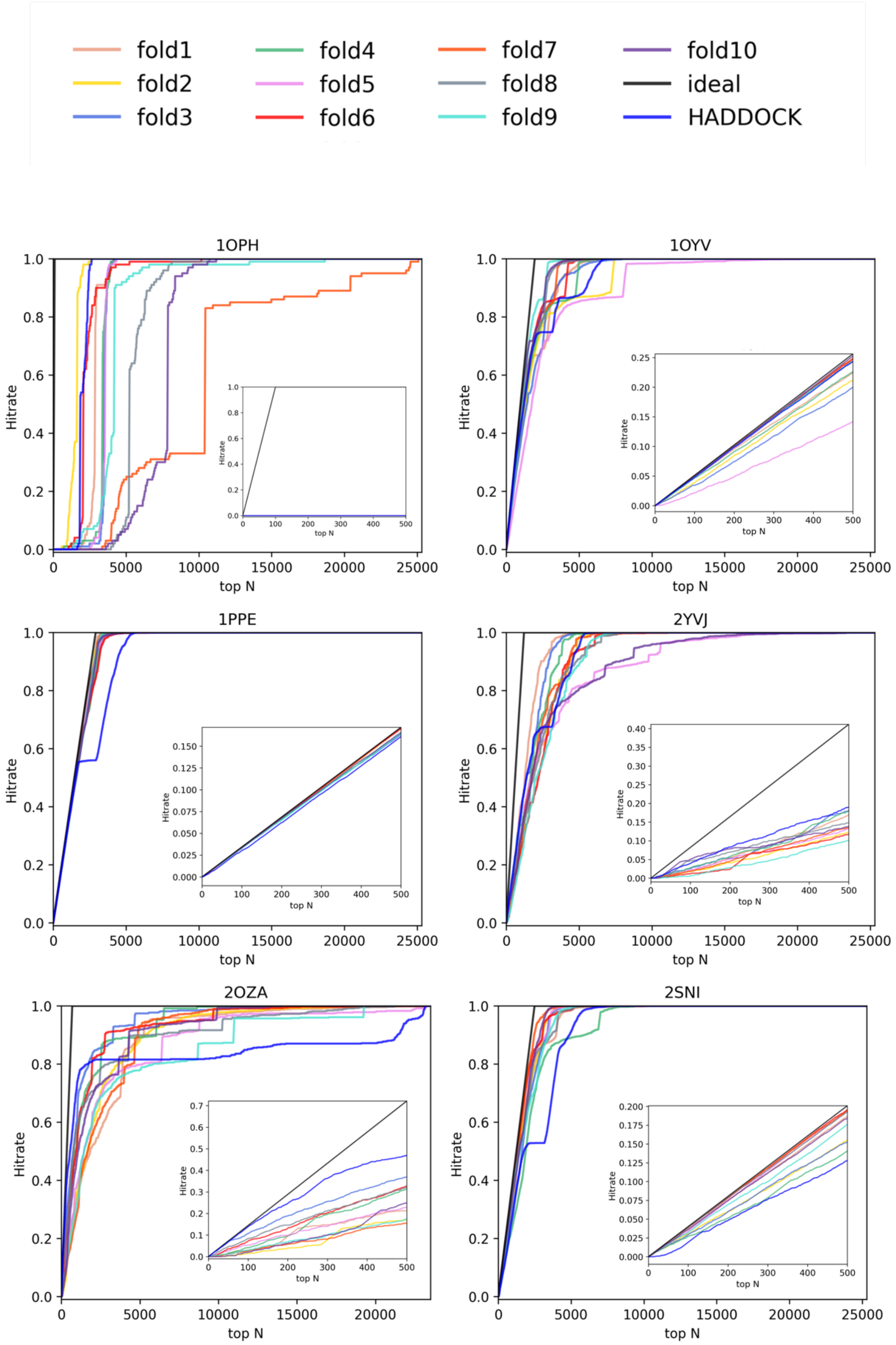
fold1
fold2
fold3
fold4
fold5
fold6
fold7
fold8
fold9
fold10
ideal
HADDOCK
1OPH
1OYV
1PPE
2YVJ
2OZA
2SNI
Hitrate
top N

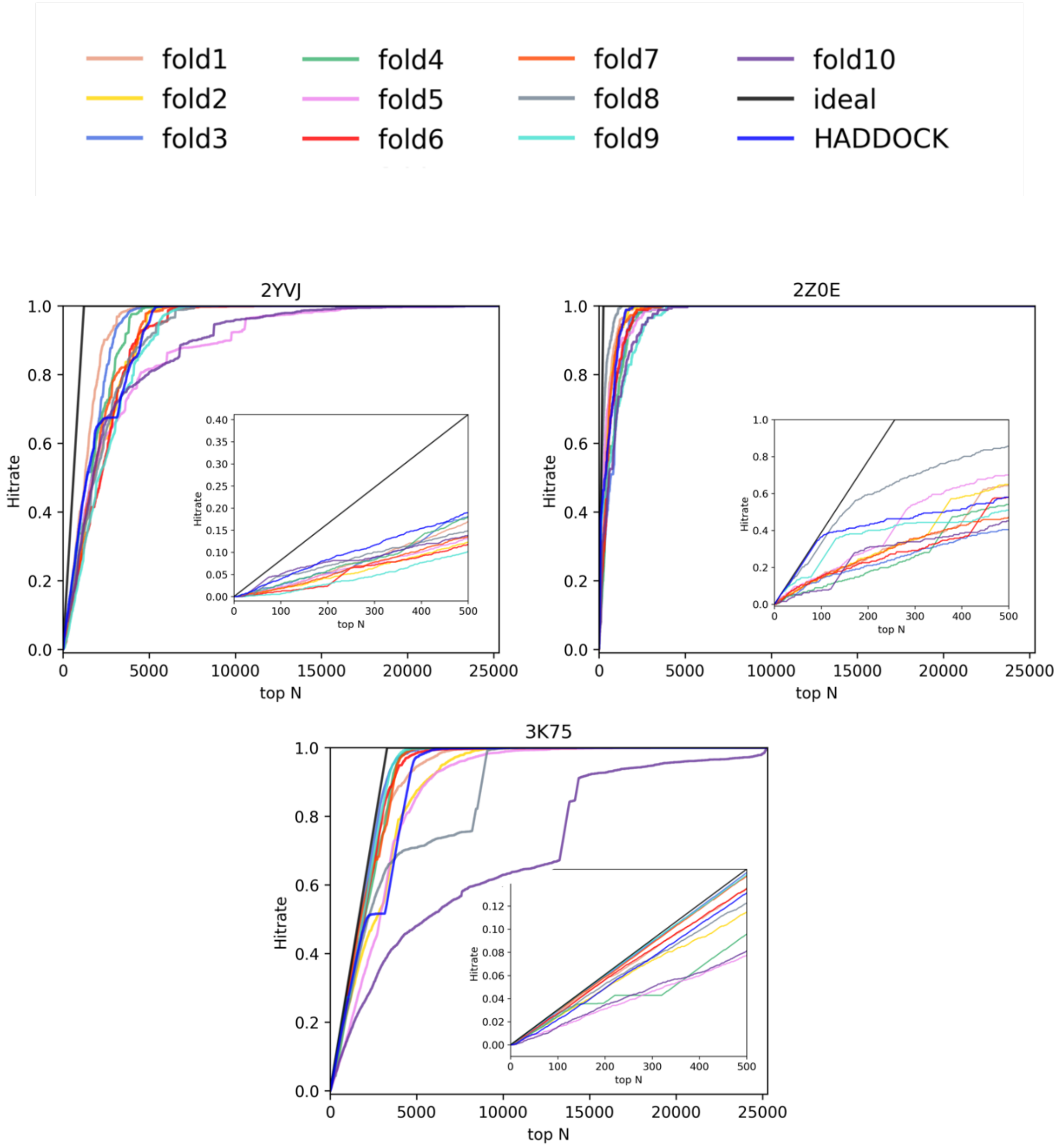
fold1
fold2
fold3
fold4
fold5
fold6
fold7
fold8
fold9
fold10
ideal
HADDOCK
2YVJ
2Z0E
3K75
Hitrate
top N

Figure S7: *Correlation plots of the measured fnat (target) and the DeepRank-GNN score (prediction) on the BM5 test dataset (it0/it1/itw). The colour code provides indications on the number of models associated with a plot area. The Spearman (rank) correlation is provided for each complex.*

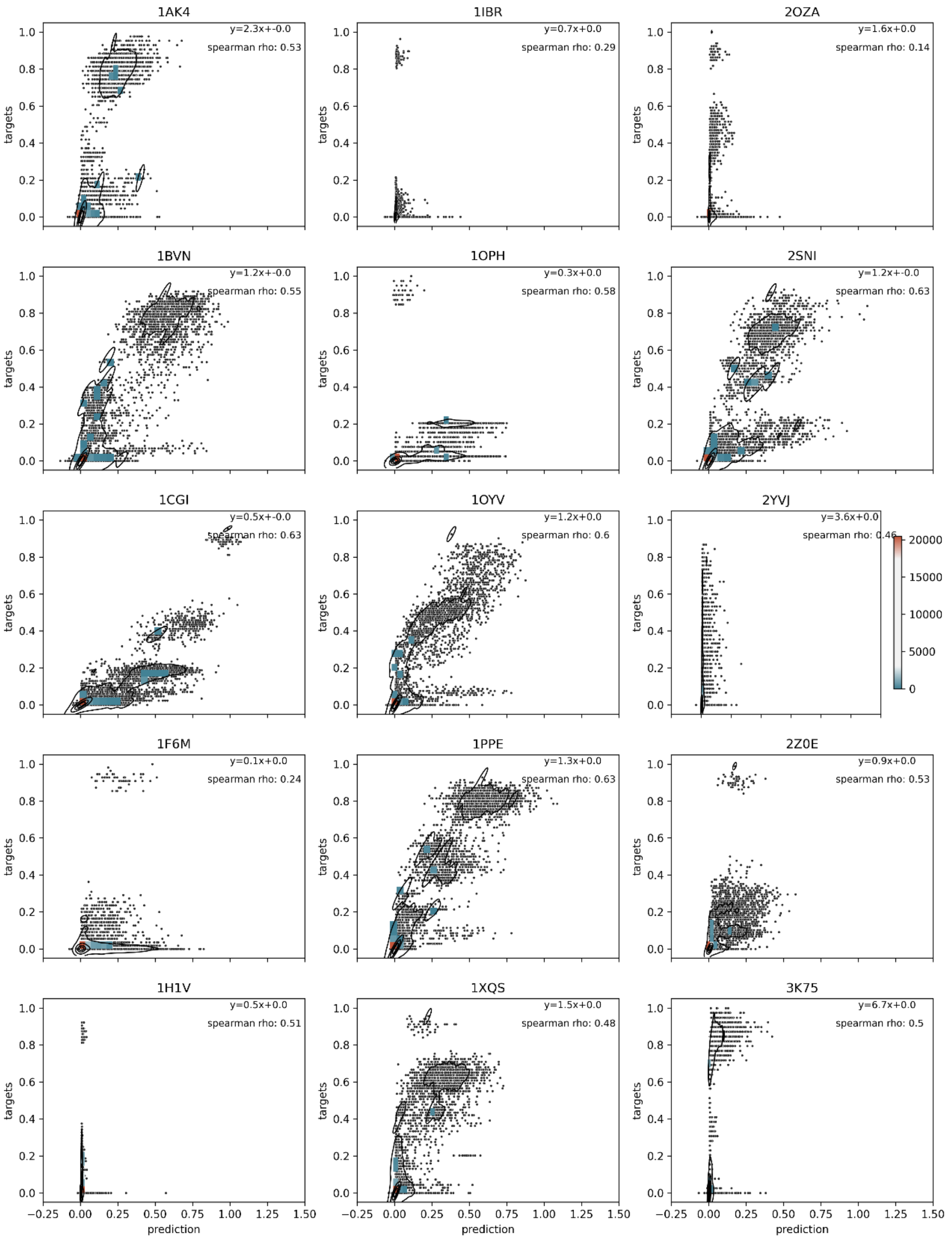

***Figure S8**: Correlation plots of the measured fnat (target) and the DeepRank-GNN score (prediction) on the BM5 test dataset (it1 and itw). The colour code provides indications on the number of models associated with a plot area. The Spearman (rank) correlation is provided for each complex.*

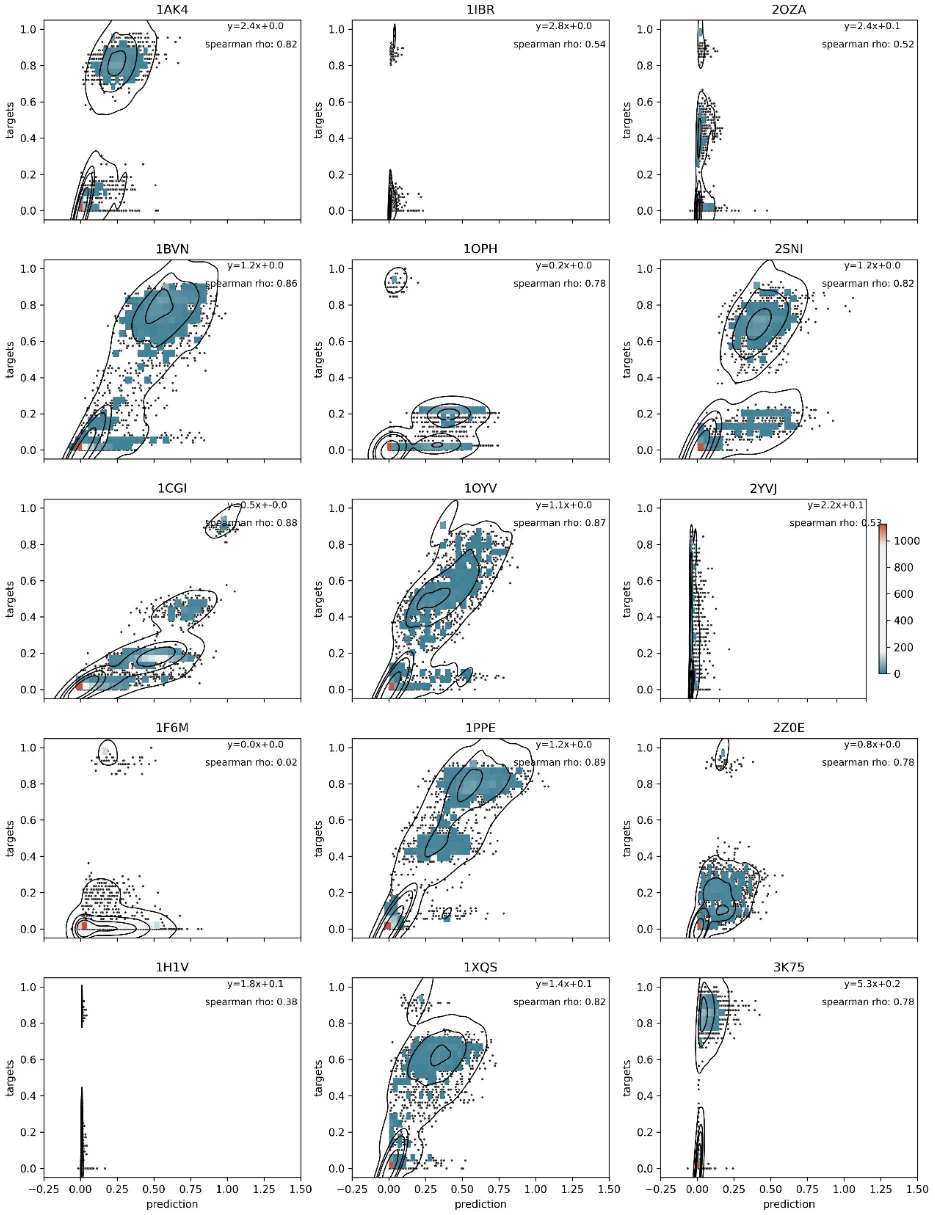

Figure S9 Comparison of the performance obtained on the CAPRI Scoreset. See legend of Fig.S4.

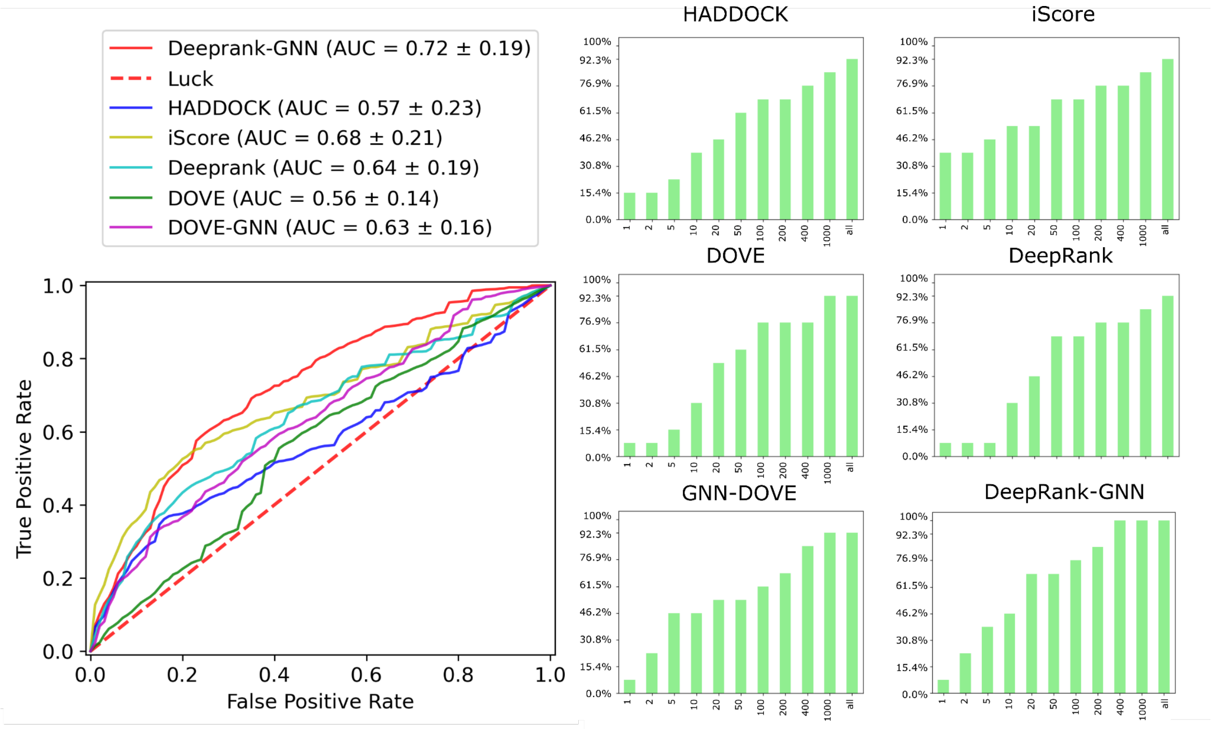

***Table S4**: Performance of the **graph** generation step of **DeepRank-GNN** on the 13 complexes (16666models) of the CAPRI score set using MPI distributed processes (4 CPUs).*

								memory	storage				
	**number of model**	**time (s)**	**time/model (s)**	**diff=(moyenne-)**	**diff*diff**	**(diff**2) * number model**	**time (min)**	**Maximum resident set size of the process during its**	**Total in MB**	**MB/model**	**diff=(moyenne-MB)**	**diff*diff**	**(diff**2) * number model**
**T29**	1979	1895,2	0,96	-0,31	0,0933	184,7	31,59	1080508	297	0,15	-0,01	0,0001	0,28
**T30**	1148	377,3	0,33	0,32	0,1046	120,1	6,29	1079676	118	0,10	0,04	0,0012	1,43
**T32**	599	499,8	0,83	-0,18	0,0332	19,9	8,33	1077288	105	0,18	-0,04	0,0014	0,83
**T35**	497	454,9	0,92	-0,26	0,0693	34,4	7,58	1074596	77	0,15	-0,02	0,0003	0,14
**T37**	1364	615,5	0,45	0,20	0,0403	55,0	10,26	1076640	184	0,13	0,00	0,0000	0,01
**T39**	1295	938,8	0,72	-0,07	0,0053	6,9	15,65	1076396	179	0,14	-0,00	0,0000	0,00
**T40**	1987	2381,3	1,20	-0,55	0,2984	593,0	39,69	1076640	288	0,14	-0,01	0,0000	0,09
**T41**	1101	321,3	0,29	0,36	0,1298	142,9	5,35	1074952	136	0,12	0,01	0,0002	0,23
**T46**	1570	701,0	0,45	0,21	0,0423	66,4	11,68	1077412	213	0,14	0,00	0,0000	0,01
**T47**	1015	319,0	0,31	0,34	0,1141	115,8	5,32	1085612	132	0,13	0,01	0,0001	0,07
**T50**	1447	1259,9	0,87	-0,22	0,0478	69,1	21,00	1081640	205	0,14	-0,00	0,0000	0,02
**T53**	1360	680,9	0,50	0,15	0,0229	31,2	11,35	1081852	204	0,15	-0,01	0,0001	0,19
**T54**	1304	423,5	0,32	0,33	0,1071	139,7	7,06	1074460	164	0,13	0,01	0,0002	0,20
	**Total**	**Average time per model (s)**				**standard deviation s (per model)**			**average MB (per model)**				**standard deviation MB (per model)**
	**16666**	**0,65**				**0,31**			**0,14**				**0,01**

__Table S5__: Performance of the __grids__ generation step of __DeepRank__ on the 13 complexes (16666models) of the CAPRI score set using MPI distributed processes (4 CPUs) with no rotation of the input model.

								memory	storage				
	number of model	time (s)	time/model (s)	diff=(moyenne-time/model)	diff*diff	(diff**2) * number model	time (min)	Maximum resident set size of the process during its lifetime, in	Total in MB	MB/model	diff=(moyenne-MB)	diff*diff	(diff**2) * number model
**T29**	1979	31495	15,91	-3,51	12,35	24450	525	267880	7168	3,62	-0,55	0,3024	598,5
**T30**	1148	10507	9,15	3,25	10,54	12105	175	200200	3072	2,68	0,40	0,1569	180,2
**T32**	599	9923	16,57	-4,17	17,36	10397	165	226952	2048	3,42	-0,35	0,1203	72,1
**T35**	497	9742	19,60	-7,20	51,87	25778	162	232060	1024	2,06	1,01	1,0237	508,8
**T37**	1364	15682	11,50	0,90	0,81	1111	261	207764	4096	3,00	0,07	0,0048	6,5
**T39**	1295	20193	15,59	-3,19	10,20	13206	337	228060	4096	3,16	-0,09	0,0082	10,7
**T40**	1987	24196	12,18	0,22	0,05	98	403	242236	6144	3,09	-0,02	0,0004	0,8
**T41**	1101	9213	8,37	4,03	16,26	17897	154	197180	3072	2,79	0,28	0,0795	87,5
**T46**	1570	16685	10,63	1,77	3,14	4931	278	214740	5120	3,26	-0,19	0,0357	56,1
**T47**	1015	9194	9,06	3,34	11,17	11333	153	199948	3072	3,03	0,05	0,0021	2,1
**T50**	1447	24763	17,11	-4,71	22,22	32150	413	237288	5120	3,54	-0,47	0,2174	314,5
**T53**	1360	14393	10,58	1,82	3,30	4488	240	206192	4096	3,01	0,06	0,0036	4,9
**T54**	1304	10667	8,18	4,22	17,80	23216	178	204936	3072	2,36	0,72	0,5131	669,1
	**Total**	**Average time per model (s)**				**standard deviation s (per model)**			**average MB (per model)**				**standard deviation MB (per model)**
	**16666**	**12,40**				**3,30**			**3,07**				**0,39**

***Table S6**: Performance of the **grids** generation step of **DeepRank** on the 13 complexes (16666models) of the CAPRI score set using MPI distributed processes (4 CPUs) with 5 rotation of the input model, i.e. 6 orientation per model in total.*

								memory	storage				
	**number of model**	**time (s)**	**time/model (s)**	**diff=(moyenne-time/model)**	**diff*diff**	**(diff**2) * number model**	**time (min)**	**Maximum resident set size of the process during its**	**Total in MB**	**MB/model**	**diff=(moyenne-MB)**	**diff*diff**	**(diff**2) * number model**
**T29**	1979	50674	25,6	-1,66	2,77	5483	845	283284	43008	21,73	-2,19	4,8114	9521,7
**T30**	1148	26084	22,7	1,22	1,49	1708	435	210112	18432	16,06	3,48	12,1310	13926,3
**T32**	599	23011	38,4	-14,47	209,51	125496	384	239180	13312	22,22	-2,69	7,2092	4318,3
**T35**	497	21927	44,1	-20,18	407,13	202343	365	227408	11264	22,66	-3,13	9,7674	4854,4
**T37**	1364	31326	23,0	0,97	0,95	1297	522	223444	24576	18,02	1,52	2,3138	3156,0
**T39**	1295	34766	26,9	-2,91	8,44	10929	579	236760	26624	20,56	-1,02	1,0412	1348,3
**T40**	1987	40965	20,6	3,32	11,05	21964	683	249468	39936	20,10	-0,56	0,3135	623,0
**T41**	1101	24444	22,2	1,74	3,03	3332	407	213892	19456	17,67	1,87	3,4875	3839,8
**T46**	1570	32386	20,6	3,31	10,98	17235	540	206932	30720	19,57	-0,03	0,0008	1,2
**T47**	1015	24895	24,5	-0,59	0,34	348	415	201952	19456	19,17	0,37	0,1371	139,1
**T50**	1447	39310	27,2	-3,23	10,40	15052	655	246148	29696	20,52	-0,98	0,9678	1400,4
**T53**	1360	29363	21,6	2,35	5,53	7516	489	213712	25600	18,82	0,72	0,5115	695,6
**T54**	1304	19854	15,2	8,72	75,97	99059	331	211440	23552	18,06	1,48	2,1826	2846,1
	**Total**	**Average time per model (s)**				**standard deviations (per model)**			**average MB (per model)**				**standard deviation MB (per model)**
	**16666**	**23,94**				**5,54**			**19,54**				**1,67**

Table S7: *Comparison of the computational performance of DeepRank-GNN and DeepRank in the training/evaluation phase using MPI distributed processes (4 CPUs). 80% of the 16666 CAPRI models fall into the training set, 20% into the evaluation set.*

	**Number of epochs**	**data augmentation**	**Total elapsed time (in seconds)**	**Total elapsed time (in min)**	**Average elapsed time per epoch (in min)**	**Maximum resident set size of the process during its lifetime, in Kbytes**	**Maximum resident set size of the process during its lifetime, in Gbytes**
**deeprank-gnn**	10	None	3458	57,6	5,76	1222200	1,17
**deeprank (cnn)** grid size= (30,30,30)		None	85752	1429,2	142,92	2106532	2,01
		5	379652	6327,5	632,75	2211720	2,11

Table S8: *DeepRank and DeepRank-GNN default features. The residue-level features highlighted in bold characters have been considered to train DeepRank and DeepRank-GNN in the comparative study detailed in section 3.3*

	Name of the features	Number of parameters
**DeepRank**	AtomicFeature	6
	**FullPSSM**	**40**
	**PSSM_IC**	**2**
	**BSA**	**2**
	**ResidueDensity**	**14**
	atomicdensities	8
	Total	72
**DeepRank-GNN**	**type**	**20**
	**charge**	**1**
	**polarity**	**4**
	**BSA**	**1**
	**PSSM**	**20**
	**cons**	**1**
	**ic**	**1**
	**Total**	**48**
